# Using AI to Detect Psychosis Relapse: Scoping Review

**DOI:** 10.2196/92192

**Published:** 2026-06-16

**Authors:** Lorenzo Ghelfi, Jack Healy, Francesco Piacenza, Ian French, Nicholas McNamara, Khyber Afridi Rabbi, Benjamin Bond, Emma O'Hora, Darren Roddy, Moyyad Kamali, Sudipto Das, Sandra Anna Just, Enrico Tedeschi, Musarrat Hussain, Karl Øyvind Mikalsen, Stefan Kaiser, Giacomo Cecere, Sanne Koops, Janna de Boer, Elysie Nguyen, Emre Bora, Wolfram Hinzen, Philipp Homan, Iris E Sommer, David Cotter, Mary Cannon, John Paul Lyne

**Affiliations:** 1 Department of Psychiatry, Royal College of Surgeons in Ireland, Smurfit Building, Beaumont, Dublin, Co Dublin, D09 YD60, Ireland, 353 0832026617; 2 University Hospital Waterford, Waterford, Munster, Ireland; 3 Health Service Executive, Leinster, Ireland; 4 Trinity College Dublin, Dublin, Leinster, Ireland; 5 Ennis Hospital, Ennis, Ireland; 6 FutureNeuro Research Centre, RCSI University of Medicine and Health Sciences, Dublin, Co Dublin, Ireland; 7 Newcastle Hospital, Greystones, Co Wicklow, Ireland; 8 UiT The Arctic University of Norway, Tromsø, Norway; 9 The Norwegian Centre for Clinical Artificial Intelligence, University Hospital of North-Norway, Trømso, Norway; 10 Department of Psychiatry, Geneva University Hospitals, Geneva, Switzerland; 11 University of Zurich, Zurich, Switzerland; 12 University of Groningen, Groningen, The Netherlands; 13 Karakter, Child and Adolescent Psychiatry, Nijmegen, The Netherlands; 14 Global Alliance of Mental Illness Advocacy Networks-Europe, Brussels, Brussels Capital, Belgium; 15 Department of Psychiatry, Faculty of Medicine, Dokuz Eylul University, Izmir, Turkey; 16 Department of Translation and Language Sciences, Universitat Pompeu Fabra, Barcelona, Spain

**Keywords:** schizophrenia, psychosis, relapse, artificial intelligence, AI, machine learning, deep learning, digital phenotyping, scoping review

## Abstract

**Background:**

Psychotic disorder represents a leading cause of disability worldwide, and relapse in psychosis is common. Artificial intelligence (AI) is increasingly recognized as a method that could aid clinical monitoring for individuals experiencing psychosis.

**Objective:**

This review aims to map the existing literature on AI-based approaches—including machine learning, deep learning, and natural language processing—used to detect relapse in individuals with psychotic disorders.

**Methods:**

A systematic search strategy was conducted on PubMed, PsycINFO, and Embase up to January 7, 2026. Observational studies, randomized controlled trials, and quasi-experimental studies that used AI methods to detect relapse in psychosis were eligible for inclusion. Screening and data extraction procedures were conducted by at least 2 reviewers working independently. Findings were extracted, charted, and described using narrative synthesis based on data extraction and consensus meetings with the research team. The scoping review was prospectively registered with the Open Science Framework.

**Results:**

Relevant studies identified (N=10) included the use of digital tools such as smartphone- and smartwatch-based monitoring, ecological momentary assessment tools, social media activity, and internet searches. Digital phenotyping via smartphones and wearables emerged as the most common method for data collection. The efficacy of AI models varied with sensitivity (or recall) ranging from 0.25 to 0.77 and specificity (or precision) ranging from 0.06 to 0.88. The reported area under the receiver operating characteristic curve for models ranged from 0.63 to 0.78. AI models were heterogeneous across studies, and most study findings were not replicated.

**Conclusions:**

This scoping review highlights both the promise and the current limitations of AI in psychosis relapse detection. Passive digital phenotyping research in the detection of psychosis relapse has progressed, and personalized approaches with individual-level modeling show promise; however, further studies need to include larger numbers of participants and should incorporate methods such as large language models. Future studies will require large collaborations aimed at delivering AI methods for use in real-world clinical practice.

## Introduction

Psychotic disorders affect approximately 0.3% to 1% of the global population and represent one of the leading causes of disability worldwide [1]. While the majority of patients with psychotic disorders achieve symptomatic remission following a first-episode psychosis, up to 80% of patients experience at least 1 episode of relapse within 5 years [2,3]. Prognosis can worsen following each relapse [4], and clinical monitoring is crucial to prevent relapses; however, this is resource-intensive [5] and presents a challenge in resource-limited settings.

Recent advancements in artificial intelligence (AI), spanning machine learning, speech and natural language processing algorithms, and sensing technology, offer new opportunities to detect psychotic symptoms remotely [6,7]. Passive sensors from smartphones measuring behavioral data provide widespread, noninvasive methods to track mental health symptom trajectories [8-11], an approach known as digital phenotyping [12,13]. Digital phenotyping typically involves collecting large quantities of health and behavioral data from service users via smartphones and wearables [14]. Sensors within these devices collect data through photoplethysmogram sensors, GPS sensors, accelerometers, gyroscopes, and light and sound sensors. These sensors, along with smartphone usage metadata, can provide a wide array of information, including data on heart rate, peripheral oxygen saturation, sleep duration, distance traveled, social media use, and sedentary behaviors [15]. The use of smartphones and wearable devices is rapidly increasing in society in general, as well as among individuals with psychotic disorders [16].

In addition to passive data sensing, several active methods (requiring the participation of service users) to assess mental health have been developed, including methods such as ecological momentary assessment (EMA) and speech-based input, which can be used to detect symptom relapse using machine learning techniques [17-19]. In contrast to standard outpatient care, these methods allow for real-time monitoring of symptoms and are not subject to recall bias.

Machine learning refers to computational methods that learn patterns from data to improve performance on specific tasks, such as using pattern recognition for classification, forming a core component of AI [20]. Within AI, language can be analyzed through spoken language processing (SLP), which focuses on acoustic and paralinguistic features (eg, prosody, phonetics, and emotion), and natural language processing (NLP), which analyzes textual features, such as lexical content, syntax, and coherence [21]. NLP often relies on transcripts produced by automatic speech recognition, an SLP tool [22].

AI methods, encompassing machine learning and deep learning (a subfield of machine learning), are inherently described as “data-hungry,” meaning they require large quantities of data to reliably identify patterns [23,24]. Digital phenotyping, which provides the scale and granularity of data needed, has the potential to work in synergy with AI methods to develop accurate and timely relapse-detection models.

Over the last decade, there has been increasing research on AI as a method for detecting psychosis relapse, as well as relapse in other mental illnesses.

However, most existing reviews have examined how AI methods could identify which patients with psychotic disorders are at higher relapse risk, using mostly static clinical, neuroimaging, and genetic data [25,26]. Our primary research aim in this study is to extend this knowledge and map the literature in this area to determine which AI methods have been studied to detect psychosis relapse in real-time, which is up to date, reflecting a rapidly evolving field. A secondary aim is to provide a narrative synthesis of the efficacy reported in each study.

## Methods

### Study Design

A scoping review was conducted following the framework proposed by Arksey and O’Malley [27]: (1) identifying the research question; (2) identifying relevant studies; (3) selecting studies; (4) charting of data; (5) collating, summarizing, and reporting results; and (6) consulting with stakeholders.

### Identifying the Research Question

Given that this area is evolving, we used scoping review methodology to identify AI methods previously studied, as well as to determine gaps in the literature. We developed our research questions following consensus meetings among the research group. Our aim was to identify AI methods that have been studied to detect psychosis relapse. The research question has a broad focus, given that there is likely to be substantial heterogeneity across AI models used in different studies to detect psychosis relapse.

Our primary outcome of interest was the relapse of psychosis, defined as a clinically significant worsening of psychotic symptoms. Relapse was identified within studies through changes in validated psychometric scores (eg, Positive and Negative Syndrome Scale and Brief Psychiatric Rating Scale), as well as through the use of proxy measures for relapse, including psychiatric hospital admission, emergency department visits, and medication adjustments (initiation, changes, or dose increases). No a priori restriction was placed on the AI tool modality used to detect the relapse of psychosis.

### Identifying Relevant Studies Using a Systematic Search Strategy

The scoping review was conducted following the PRISMA-ScR (Preferred Reporting Items for Systematic Reviews and Meta-Analyses Extension for Scoping Reviews) guidelines (Checklist 1). The scoping review protocol was prospectively registered on the Open Science Framework (YKQMG). Covidence software was used to manage the systematic search and screening process [28].

We searched PubMed, PsycINFO, and Embase up to January 7, 2026, with no restrictions on the publication date. The search strategy combined keywords and MeSH (Medical Subject Headings) terms. The search strategy included terms (Textbox 1) such as:

[schizophreni* OR schizoaff* OR psychotic OR psychosis] AND [“artificial intelligence” OR “AI” OR “machine learning” OR “deep learning” OR “digital phenotyping” OR “language processing” OR “SLP” OR “NLP”]

We performed forward and backward (“snowballing”) citation tracking on key articles identified during our search to augment our initial search.

Textbox 1. **Search strings for search strategy.**

**PubMed**

(schizophreni* OR schizoaff* OR psychotic OR psychosis)

AND

("artificial intelligence" OR AI OR "machine learning" OR "deep learning" OR "digital phenotyping" OR "language processing" OR SLP OR NLP)

**Embase**

('schizophrenia'/exp OR schizophreni* OR schizoaff* OR 'psychosis'/exp OR psychotic OR psychosis)

AND

('artificial intelligence'/exp OR 'artificial intelligence' OR ai OR 'machine learning'/exp OR 'machine learning' OR 'deep learning'/exp OR 'deep learning' OR 'digital phenotyping' OR 'natural language processing'/exp OR 'natural language processing' OR (language NEXT/1 processing) OR slp OR nlp)

**PsycINFO**

((schizophreni* OR schizoaff* OR psychotic OR psychosis))

AND

(("artificial intelligence" OR AI OR "machine learning" OR "deep learning" OR "digital phenotyping" OR "natural language processing" OR NLP OR SLP OR (language N1 processing)))

### Study Selection

Following the removal of duplicate studies, each abstract was independently reviewed on Covidence by at least 2 reviewers from a team of 8 reviewers (LG, JH, BB, NM, IF, FP, EO, and KAR). Reviewer pairs were not preassigned, allowing for flexible allocation across the screening process. The inclusion criteria were as follows: (1) observational studies, randomized controlled trials, and quasi-experimental studies; (2) studies focusing on psychotic disorders; and (3) studies reporting AI-based methods to detect relapse of psychosis. The exclusion criteria for the search were as follows: (1) non–English language articles, (2) review articles, (3) conference abstracts and editorials, (4) mixed studies of patients with psychotic and nonpsychotic conditions unless findings were reported separately for patients with psychotic disorders, (5) studies focusing on clinical high-risk populations, (6) studies on animal models, (7) studies using traditional statistical methods without machine learning or deep learning components, (8) studies without full-text articles available online, and (9) studies with data completely subsumed in other included studies unless a different AI detection model was investigated.

The research group met regularly (every 1‐2 wk for a 6-mo period) to resolve any discrepancies between the 2 independent researchers. Articles passing the abstract screening process underwent full-text review by 2 independent reviewers, with clear documentation of reasons for exclusion. Any disagreements were resolved through consensus and consultation during regular group meetings, with input from senior investigators.

### Extraction and Charting of Data

Data from each included paper were extracted independently by 2 reviewers using a standardized data extraction form developed a priori and piloted on a subset of articles (the data extraction template can be found in the PRISMA-ScR checklist). Extracted data included study characteristics (authors, publication year, country, and study design), participant characteristics (sample size, age, gender, and diagnosis), outcome definitions, data sources (electronic health records, mobile devices, and wearable sensors), AI model specifications (algorithm type, training or validation approach, and feature selection), and reported performance metrics, including sensitivity, specificity, positive and negative predictive values, accuracy, and area under the receiver operating characteristic curve (AUROC). We reported global performance metrics aggregated across all participants rather than individualized or patient-specific results reported in some studies. Discrepancies in data extraction were resolved by consensus, with consultation from a third reviewer when necessary.

### Collating, Summarizing, and Reporting Results

Extracted data were charted in a summary table (see “Results” section), prepared independently by 2 data extractors. Charted data were compared across the data extractors, and consensus was reached with senior investigator input for final tabulation. Results are presented using a figure and the summary tables to facilitate comparison across studies where appropriate. Considering the heterogeneity in study designs, populations, AI methodologies, and outcome definitions, as well as the novelty of the topic, the studies were deemed unsuitable for quantitative meta-analysis following consultation with our departmental statistician. Narrative synthesis was used with detailed descriptive analysis of study characteristics, methodological approaches, and reported outcomes. Risk of bias assessment was also considered and further discussed with departmental statisticians; however, this was deemed inappropriate in the context of the heterogeneous data available across papers and was not deemed essential given that a scoping review protocol was used for the study.

### Consultation With Stakeholders

Articles for inclusion were discussed by the research team at consensus meetings, and study results were reviewed by the research team. Once the data were summarized, the study results were discussed at meetings with research stakeholders, including senior authors. The synthesis of the findings and implications of the study was summarized using the consensus opinion of the research team, which included input from several research groups and a mental illness advocacy network. Consultation with AI expertise (SD) was conducted during the screening process and for the synthesis of study findings.

## Results

### Study Selection and Characteristics

**Figure 1.**
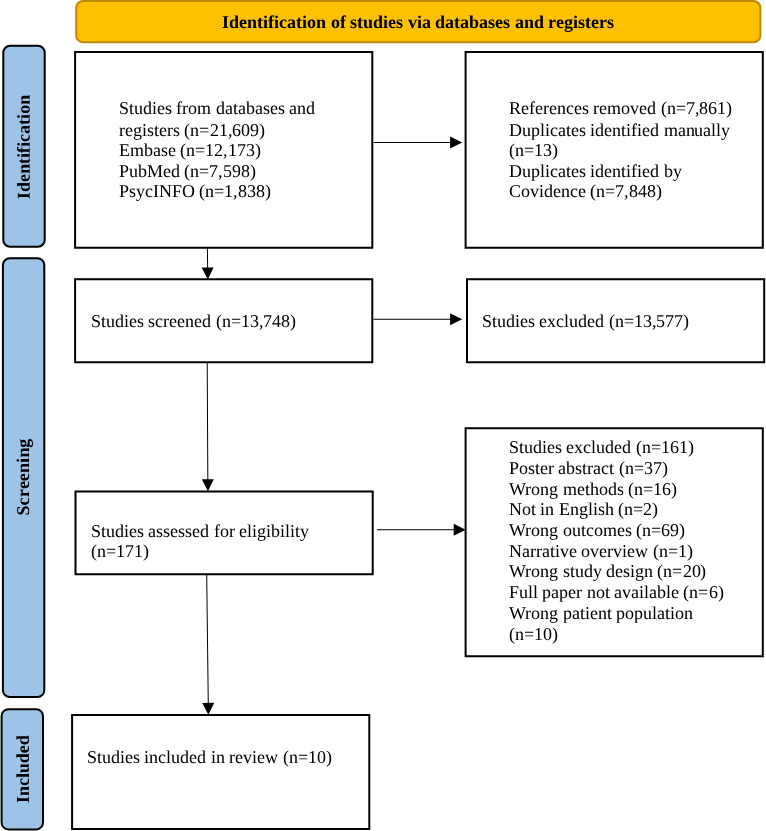
PRISMA (Preferred Reporting Items for Systematic Reviews and Meta-Analyses) flowchart.

The PRISMA (Preferred Reporting Items for Systematic Reviews and Meta-Analyses) flowchart [29] for the study is available in Figure 1. A total of 13,748 study abstracts were screened for relevance, of which 171 studies underwent full-text review, yielding 10 eligible studies published between 2019 and 2025 (Table 1) [30-39]. Sample sizes ranged from 7 to 268 total participants, and the majority of studies were conducted in the United States. No eligible studies were identified through gray literature or preprint sources.

**Table 1.** Characteristics across included studies (N=10).

Study	Country	Sample size, n	Female, n (%)	Age (y)
Adler et al [36]	United States	60 patients	25 (41.6)	Relapse group, median (IQR): 33 (23-46) Nonrelapse group, median (IQR): 40 (26-50)
Birnbaum et al [30]	United States	51 patients	15 (29.4)	Mean (SD): 23.96 (4.59)
Birnbaum et al [32]	United States	42 patients, 74 controls (116 total)	65 (56)	Mean (SD): 24.38 (5.18)
Buck et al [39]	United States	61 patients	25 (41)	Mean (SD): 37.11 (13.85)
Lamichhane et al [37]	United States	63 patients^a^	36 (57.1)	Mean (SD): 37.2 (13.7)
Nguyen et al [31]	United States	141 patients, 127 controls (268 total)	52 (36.9)	Mean (SD): 24.86 (5.49)
Tsakmaki et al [35]	Greece	7 patients (30 in validation sample)	—^c^ (53.3 in validation sample)	Mean (SD): 32.1 (7.3) Validation sample: 20‐69
Yan et al [34]	United States and Greece	10 patients^b^	4 (40)	Mean (SD): 30.60 (7.31)
Zhou et al [38]	United States	63 patients^a^	36 (57.1)	Mean (SD): 37.2 (13.7)
Zlatintsi et al [33]	Greece	10 patients^b^	4 (40)	Mean (SD): 30.60 (7.31)

a Overlapping participant samples.

b Overlapping participant samples.

c Not available.

### Data Modalities

The primary data modalities used for relapse detection included mobile and wearable device sensor data (GPS location, phone calls, SMS text messaging patterns, and accelerometer data), internet search behavior, and speech or language data. Smartphone-based monitoring was used in 6 of 10 studies. Two studies used social media platforms to detect psychosis relapse (Birnbaum et al [30] on Facebook posts and Nguyen et al [31] on multiplatform content). Other sources included internet search activity [32], smartwatch wearables [33-35], and audiovisual recordings [33].

### AI Methods

Across the 10 studies, AI methods used can be categorized into 4 groups as follows:

- Traditional supervised machine learning was present in 3 out of 10 studies, including support vector machines, random forests, and gradient boosting [30-32].
- Deep learning was present in 5 out of 10 studies, mainly involving encoder-decoder autoencoders and sequence models [33-37].
- One study used hybrid pipelines that paired unsupervised components including clustering and anomaly scores, with a supervised classifier [38].
- One study used a speech detection algorithm to generate data, which was then examined using a linear regression model [39].

A key challenge in relapse detection is the rarity of events, which leads to class imbalance. Anomaly detection, defined as the statistical and computational task of identifying observations or patterns in data that deviate significantly from an established model of normal behavior [40,41], represents a common method to handle the skewed distribution of relapse events in digital phenotyping studies [42-44]. This framework was integrated with deep learning approaches in 2 studies [33,34]. Additionally, Lamichhane et al [37] used anomaly detection as a comparison baseline for their supervised long–short-term memory approach.

Additional strategies used to manage class imbalance included restricting analysis to individuals who experienced relapse [30], and training models only on nonrelapse periods while evaluating them on relapse events [34]. As part of a temporal rebalancing strategy, Birnbaum et al [32] compared different relapse-proximal and relapse-distal window lengths (eg, 1 mo vs 1 mo, 1 mo vs 2 mo, and 1 mo vs 3 mo) and found that the 1- to 1-month window provided the best trade-off for classification. A similar time frame was analyzed by other studies [36,39]. This approach not only mitigates class imbalance in longitudinal data but also reflects the phenomenology of psychosis relapse, which often emerges gradually with an insidious onset rather than as a discrete, switch-like event.

### Performance Metrics

Reported sensitivities (or recall) ranged from 0.25 [36] to 0.77 [37], which was achieved using a personalized long–short-term memory, although precision was low in light of relapse being a rare event. Specificity ranged from 0.71 [30] to 0.88 [36]. AUROC values, when provided, ranged from approximately 0.633 to 0.779 [33,34]. The *F*_1_-scores reported ranged from 0.72 [31] to 0.9817 [35], while the *F*_2_-score ranged from 0.16 to 0.3. Performance metrics for each study are presented in Table 2.

Focusing on linguistic aspects of relapse detection, multiple lines of evidence have identified specific alterations, including the use of words related to the categories of “anger,” “death,” or “swear,” and, more generally, words pertaining to negative affect [30-32].

**Table 2.** Summary of models used and their associated performance.

Study	Modality or data source	Model	Performance	Model validation	Best predicting feature
Adler et al [36]	CrossCheck app	Encoder-decoder neural networks	Sensitivity 0.25 Specificity 0.88	Monte Carlo cross-validation with 100 iterations, plus multiple data splits (training, cross-validation, test sets)	Sleep disruption
Birnbaum et al [30]	Facebook archives	Ensemble model	Sensitivity 0.38 Specificity 0.71 PPV^a^ 0.66 NPV^b^ 0.44	90% training or 10% testing split approach	Linguistic features, that is, negative affect
Birnbaum et al [32]	Internet-search data	SVM^c^ RF^d^ GB^e^	AUROC^f^ 0.71 (0.16 SD) AUROC 0.69 (0.09 SD) AUROC 0.71 (0.10 SD)	Training or validation data splits mentioned	Reduced length of search queries during relapse periods
Buck et al [39]	CrossCheck app	Regression models	N/A^g^ Outgoing call duration (h): β=−0.019; *P*=.03 Number of incoming SMS: β=−2.228; *P*=.01 Number of outgoing SMS: β=−2.435; *P*=.02	No	Outgoing SMS
Lamichhane et al [37]	CrossCheck app	LSTM^h^-based deep learning model^i^ Encoder-decoder neural networks Fusion model	Recall 0.77**,** *F*_2_-score 0.21, precision 0.06 *F*_2_-score 0.16 Recall 0.56, *F*_2_-score 0.3, precision 0.1	LOPO^j^ cross-validation with nested cross-validation for hyperparameter tuning	Conversation and volume
Nguyen et al [31]	Social media	Logistic regression^i^ RF SVM MLP^k^	Average *F*_1_-score 0.72 (SD 0.07), accuracy of 0.81 (SD 0.08), and AUROC of 0.749 (SD 0.06)	80:20 train-test split with 5-fold stratified cross-validation for hyperparameter tuning	Average post readability
Tsakmaki et al [35]	Wearable PPG^l^-derived HRV^m^ using Smartwatch wearable (Samsung Gear S3 [Samsung Electronics])	Personalized LSTM^i^ Transformer model Traditional ML^n^ models (kNN^o^, DT^p^, RF, GB, MLP)	Mean *F*_1_-score 0.9817 Mean recall 0.9897 Mean precision 0.9770	Repeated 80:20 train-splits (100 iterations) Friedman-Nemenyi tests; LOPO External validation on independent 30-patient cohort *F*_1_-score: 0.784, recall: 0.851, precision: 0.758	N/A
Yan et al [34]	Smartwatch wearable (Samsung Gear S3; e-Prevention)	2D convolutional autoencoder and unsupervised clustering (k-means/GMM^q^)	PR-AUC^r^ 0.716, ROC-AUC^s^ 0.633, harmonic mean 0.672 (sleep-only); silhouette 0.18	5-fold cross-validation	Sleep, HRV
Zhou et al [38]	CrossCheck	Clustering: GMM, partition around medoids Classification: balanced RF	*F*_2_-scores (all features): 0.23 (0.063/0.662)	LOPO cross-validation with nested cross-validation	Mobility metrics
Zlatintsi et al [33]	Smartwatch wearable (Samsung Gear S3; e-Prevention dataset); audiovisual interviews	Four different autoencoder architectures were examined: Transformers Fully connected neural networks Convolution neural networks^i^ Gated recurrent units	ROC-AUC 0.779	5-fold cross-validation with 60%/20%/20% train/validation/test splits	Combination of accelerometer, gyroscope, and heart rate

a PPV: positive predictive value.

b NPV: negative predictive value.

c SVM: support vector machine.

d RF: random forest.

e GB: gradient boosting.

f AUROC: area under the receiver operating characteristic curve.

g N/A: not applicable.

h LSTM: long–short-term memory.

i Best-performing model.

j LOPO: leave-one-patient-out.

k MLP: multilayer perceptron.

l PPG: photoplethysmography.

m HRV: heart rate variability.

n ML: machine learning.

o kNN: k-nearest neighbors.

p DT: decision tree.

q GMM: Gaussian mixture model.

r PR-AUC: precision-recall area under the curve.

s ROC-AUC: receiver operating characteristic area under the curve.

### Model Validation

Model validation approaches varied considerably across studies. As most studies included modest sample sizes with few relapse events, internal validation was common. Adler et al [36] used Monte Carlo cross-validation with 100 iterations alongside multiple data splits (training, cross-validation, and test sets). Leave-one-patient-out cross-validation, often with nested cross-validation for hyperparameter tuning, was implemented by Lamichhane et al [37], Zhou et al [38], and Tsakmaki et al [35]. Traditional train-test splits were used by several studies: Nguyen et al [31] used an 80:20 split with 5-fold stratified cross-validation for hyperparameter optimization, while Birnbaum et al [30,32] used a 90:10 split and other training/validation splits, respectively. Zlatintsi et al [33] and Yan et al [34] both implemented 5-fold cross-validation with systematic data partitioning. Only 1 study [32] validated findings in an independent external cohort (N=30), demonstrating a marked reduction in performance (recall dropped from 0.9897 to 0.851 in the validation sample).

## Discussion

### Summary of Study Findings

This study presents a scoping review of AI methods used to detect relapse of psychosis. We identified 10 relevant articles that combined different methodologies to detect relapse of psychosis, including smartphone- and smartwatch-based monitoring, EMAs or diaries, social media activity, internet searches, and audiovisual recordings of patients with psychosis.

Digital phenotyping, consisting of active, passive, or combined data collection, has emerged as the most common framework for collecting data used in the detection of psychosis relapse. Several mobile phone apps have been developed and adopted to detect relapse through digital phenotyping, including CrossCheck [36-39], MindLAMP [43,44], Beiwe [42,43], and SleepSight [45]; however, to our knowledge, only CrossCheck has been paired with AI methods. The specific AI models deployed used supervised machine learning, deep learning, and computational statistical methods, including anomaly detection algorithms, automated time-series analysis, and associational pattern detection models.

Overall, there was considerable heterogeneity noted in terms of the efficacy of detection results, with sensitivities ranging from 0.25 to 0.77 and specificities from 0.06 to 0.88. AUROC values generally indicated moderate discrimination (0.633‐0.779). Where reported, precision was low (0.06 in Lamichhane et al [37] and 0.063 in Zhou et al [38], drawing from the same CrossCheck population), reflecting the difficulty of modeling relapse events against a preponderance of nonrelapse observations.

This review illustrates emerging but heterogeneous trends in the use of AI models as predictors of psychosis relapse. Small sample sizes and widespread variations in methodology limit generalizability, but these preliminary results suggest the need to explore this approach through further studies.

While the significant heterogeneity of the included studies precludes any definitive conclusion, several promising directions emerged from this review. Passive digital phenotyping has been the most widely studied method for long-term monitoring and demonstrates good potential for detecting psychosis relapse (eg, recall 0.77 in the study by Lamichhane et al [37]). Compared to active digital phenotyping like EMA, passive sensing offers a viable and effective method with lower participant burden. Wearable-based physiologic monitoring, as used in the e-Prevention cohort [33-35], represents a complementary approach with the advantage of capturing continuous signals, such as heart rate variability, independently of smartphone engagement. Overall, personalized models achieved the strongest performance in this review, suggesting individual-level modeling is a promising direction for future work.

### Clinical Implications

The adoption of AI in clinical practice will depend on multiple factors: access to technical resources, cultural readiness to engage with digital tools and AI among clinicians and service users, and health system infrastructure. Given that approximately 80% of people in high-income countries own a smartphone [46], smartphone-based digital phenotyping represents one of the most practical approaches to implementation.

Encouragingly, good compliance has been reported in related contexts: Busk et al [47] observed 91% adherence to the Monsenso system in individuals with bipolar disorder, though this platform has not yet been adapted to psychosis relapse detection. However, a systematic review has cautioned that such compliance rates may be inflated due to selection bias, particularly given that participants consenting to research involvement might be more willing to engage with digital monitoring [15]. Furthermore, monitoring behavior itself may be influenced by a Hawthorne effect, potentially limiting generalizability [15].

In a recent mixed methods study [48], stakeholders, including young people, viewed AI-informed mobile mental health apps as promising tools for prevention and self-support, particularly when they offer tailored feedback, personalized interventions, and user-friendly designs. However, in the same study, participants emphasized the need for transparency, data privacy, and user control over AI-driven features. Similarly, a qualitative study on passive sensing to detect relapse in individuals with psychosis highlighted that some participants felt uneasy about continuous monitoring, especially location tracking, which they perceived as intrusive and threatening to their privacy [49].

A key consideration for future implementation will be the interpretability of AI models. Computational anomaly detection approaches [42,43] offer relatively transparent insights into which behavioral features deviate before relapse, whereas most deep learning models remain “black boxes.” None of the included studies adopted explainable AI techniques, such as Shapley Additive Explanations or Local Interpretable Model-Agnostic Explanations, which attribute a model’s prediction to specific input features [50-52], with the exception of 1 study by Nguyen et al [31]. Explainability is crucial in clinical psychiatry to ensure that clinicians and service users trust the reasons why a system might flag elevated relapse risk. Integrating explainable AI frameworks alongside model development will enhance transparency, user confidence, and regulatory readiness without sacrificing predictive accuracy.

A lack of interpretability can also contribute to the practical challenges frequently observed in digital monitoring systems such as alarm fatigue. Continuous monitoring systems may generate frequent alerts, including false alarms, due to noise in behavioral data or transient fluctuations not related to relapse [53]. Excessive alarm risk can desensitize clinicians and service users, likely reducing engagement with the system [54]. Adaptive threshold–based alarm systems, which adjust detection limits according to individual behavioral baselines, may help reduce false alarms while maintaining sensitivity to true relapse signals [53]. Future work should consider methods that optimize alarm specificity to minimize alarm burden while preserving clinical sensitivity.

Finally, a related challenge concerns the reliability of the relapse labels used to evaluate these models. Birnbaum et al [30] examined false alarms in detail by performing clinical chart reviews, highlighting how many of these signals actually corresponded to clinical deterioration. The results suggested that model performance may be underestimated, underscoring the need to consider complementary methods to monitor relapse, clinical deterioration, and medication adherence [55].

### Methodological Considerations

The reported performance metrics should be interpreted cautiously in light of several methodological challenges, with class imbalance representing a ubiquitous concern across studies. For example, while 20 out of 63 patients from the CrossCheck cohort experienced relapse, only 3.7% of the observed data fell within near-relapse windows. Several studies adopted strategies to address this, including anomaly detection frameworks [34,36], one-class classification [30], and temporal rebalancing through relapse-proximal windowing; however, precision remained low where reported [37,38], indicating that a high proportion of model-generated alerts would likely be false positives in clinical practice.

Validation strategies also varied significantly. As Lamichhane et al [37] noted, Wang et al [56] used random k-fold cross-validation on longitudinal data, which risks introducing temporal information leakage, whereby data collected after a given prediction time point may inadvertently inform model training, potentially inflating performance estimates [57]. In contrast, leave-one-patient-out cross-validation with sequential prediction, as implemented by Lamichhane et al [37] and Zhou et al [38], better approximates prospective clinical deployment, and notably, these studies reported comparatively modest performance.

It is also important to consider the degree of dataset overlap across the included studies. Four studies [36-39] drew from the same CrossCheck cohort, while 3 studies [33-35] used the e-Prevention cohort from Athens. As such, the included studies effectively represent a limited number of independent patient samples, which should be considered when appraising the cumulative strength of the evidence presented.

### Limitations

The findings of this scoping review should be carefully interpreted in light of numerous limitations. Overall, the main limitations across studies were modest sample sizes and marked heterogeneity in AI methodologies, data modalities, validation strategies, and reporting of performance metrics. Many studies relied on pilot or feasibility samples with short follow-up periods, which may not capture the full clinical trajectory of psychosis relapse. Additionally, most studies were conducted in high-income countries and predominantly involved younger adult samples, further limiting generalizability. Our search was limited to peer-reviewed journal articles and did not include gray literature, such as conference proceedings or preprints. While several conference abstracts were identified during screening, none of these met the criteria for inclusion.

Relapse definitions varied considerably across studies, ranging from psychometric thresholds (eg, Positive and Negative Syndrome Scale score changes) to proxy measures such as hospitalization or medication adjustments, complicating cross-study comparison of model performance. Methodologically, many studies tested multiple machine learning or deep learning algorithms on the same dataset with little justification, often in a trial-and-error fashion. This approach risks overfitting and weakens reproducibility. For feature-based data, reproducibility could be improved in future studies through transparent feature selection and stability checks using penalized regression or bootstrapped resampling.

Given that interoperability and integration with existing health systems will be important for future clinical implementation, overall, most studies identified remain at an early, exploratory stage. Based on these findings, fundamental issues such as methodological rigor, replication, and validation represent current challenges for the field.

### Future Research

There is much opportunity to further develop this field, although it should be acknowledged that research will require adequate resources and expertise. An important question for future research is whether combining these AI methods could result in more accurate detection of psychosis with higher sensitivity and specificity. This might require research groups to collaborate and share expertise to develop AI methods. In a comparable context, AI-based detection using electroencephalography data has achieved robust and reproducible performance, offering a useful benchmark for research on psychosis relapse [58]. Future research should also aim to replicate AI model findings in prospectively collected data, and studies should include larger and more diversified patient populations.

Other potential applications of the AI methods identified in this scoping review include supporting psychiatric diagnosis [59], predicting transition to psychosis among individuals with clinical high risk, and reducing clinical workloads by delivering psychometric scales to individuals with psychosis [60]. The recent emergence of large language models could contribute further to this field. Early studies indicate that large language models may be able to detect speech changes early in psychosis relapse, which could provide an efficacious approach for relapse detection [61,62].

Emerging regulations, such as the EU Artificial Intelligence Act and the European Health Data Space, are helpful in guiding the responsible use of AI in psychiatry [63]. These frameworks emphasize transparency, explainability, and secure data sharing—principles that can underpin the safe translation of AI approaches into clinical practice.

### Conclusions

AI methods, particularly when integrated with passive digital phenotyping approaches using smartphones and wearable devices, show promise in detecting relapse of psychosis. The use of personalized approaches with individual-level modeling shows the most promise based on our findings and merits further research. Nonetheless, current evidence remains preliminary, and notably, there is a lack of replication across most studies identified in this scoping review. Hence, it is difficult to recommend any particular approach for usage in clinical practice, and there are several potential barriers to implementing AI methods in real-world clinical practice. Developing this field further will require large collaborations across research groups, combining expertise across medical and nonmedical fields, while incorporating lived experience input for the development of new AI methods.

## Supplementary material

10.2196/92192 Checklist 1 PRISMA-ScR checklist.

## Abbreviations

AI: artificial intelligence
AUROC: area under the receiver operating characteristic curve
EMA: ecological momentary assessment
NLP: natural language processing
PRISMA: Preferred Reporting Items for Systematic Reviews and Meta-Analyses
PRISMA-ScR: Preferred Reporting Items for Systematic Reviews and Meta-Analyses Extension for Scoping Reviews
SLP: spoken language processing

## References

[1] Charlson FJ, Ferrari AJ, Santomauro DF (2018). Global epidemiology and burden of schizophrenia: findings from the Global Burden of Disease Study 2016. Schizophr Bull.

[2] Robinson D, Woerner MG, Alvir JM (1999). Predictors of relapse following response from a first episode of schizophrenia or schizoaffective disorder. Arch Gen Psychiatry.

[3] Alvarez-Jimenez M, Priede A, Hetrick SE (2012). Risk factors for relapse following treatment for first episode psychosis: a systematic review and meta-analysis of longitudinal studies. Schizophr Res.

[4] Henson P, Wisniewski H, Stromeyer Iv C, Torous J (2020). Digital health around clinical high risk and first-episode psychosis. Curr Psychiatry Rep.

[5] Bernardo M, Ramón Azanza J, Rubio-Terrés C, Rejas J (2006). Cost-effectiveness analysis of schizophrenia relapse prevention: an economic evaluation of the ZEUS (Ziprasidone-Extended-Use-In-Schizophrenia) study in Spain. Clin Drug Investig.

[6] Torous J, Onnela JP, Keshavan M (2017). New dimensions and new tools to realize the potential of RDoC: digital phenotyping via smartphones and connected devices. Transl Psychiatry.

[7] Insel TR (2018). Digital phenotyping: a global tool for psychiatry. World Psychiatry.

[8] Frost M, Marcu G, Hansen R, Szaántó K, Bardram JE The MONARCA self-assessment system: persuasive personal monitoring for bipolar patients.

[9] Wang R, Chen F, Chen Z StudentLife: assessing mental health, academic performance and behavioral trends of college students using smartphones.

[10] Osmani V (2015). Smartphones in mental health: detecting depressive and manic episodes. IEEE Pervasive Comput.

[11] Saeb S, Zhang M, Karr CJ (2015). Mobile phone sensor correlates of depressive symptom severity in daily-life behavior: an exploratory study. J Med Internet Res.

[12] Torous J, Kiang MV, Lorme J, Onnela JP (2016). New tools for new research in psychiatry: a scalable and customizable platform to empower data driven smartphone research. JMIR Ment Health.

[13] Mohr DC, Shilton K, Hotopf M (2020). Digital phenotyping, behavioral sensing, or personal sensing: names and transparency in the digital age. NPJ Digit Med.

[14] Benoit J, Onyeaka H, Keshavan M, Torous J (2020). Systematic review of digital phenotyping and machine learning in psychosis spectrum illnesses. Harv Rev Psychiatry.

[15] Saccaro LF, Amatori G, Cappelli A, Mazziotti R, Dell’Osso L, Rutigliano G (2021). Portable technologies for digital phenotyping of bipolar disorder: a systematic review. J Affect Disord.

[16] Firth J, Cotter J, Torous J, Bucci S, Firth JA, Yung AR (2016). Mobile phone ownership and endorsement of “mHealth” among people with psychosis: a meta-analysis of cross-sectional studies. Schizophr Bull.

[17] Ciampelli S, de Boer JN, Voppel AE (2023). Syntactic network analysis in schizophrenia-spectrum disorders. Schizophr Bull.

[18] Tan EJ, Sommer IEC, Palaniyappan L (2023). Language and psychosis: tightening the association. Schizophr Bull.

[19] Palominos C, He R, Fröhlich K (2024). Approximating the semantic space: word embedding techniques in psychiatric speech analysis. Schizophrenia (Heidelb).

[20] Jordan MI, Mitchell TM (2015). Machine learning: trends, perspectives, and prospects. Science.

[21] Young T, Hazarika D, Poria S, Cambria E (2018). Recent trends in deep learning based natural language processing [Review Article]. IEEE Comput Intell Mag.

[22] Eyben F, Scherer KR, Schuller BW (2016). The Geneva minimalistic acoustic parameter set (GeMAPS) for voice research and affective computing. IEEE Trans Affective Comput.

[23] Adadi A (2021). A survey on data‐efficient algorithms in big data era. J Big Data.

[24] Riley RD, Ensor J, Snell KIE (2025). Importance of sample size on the quality and utility of AI-based prediction models for healthcare. Lancet Digit Health.

[25] Tay JL, Htun KK, Sim K (2024). Prediction of clinical outcomes in psychotic disorders using artificial intelligence methods: a scoping review. Brain Sci.

[26] Tay JL, Ang YL, Tam WWS, Sim K (2025). Accuracy of machine learning methods in predicting prognosis of patients with psychotic spectrum disorders: a systematic review. BMJ Open.

[27] Arksey H, O’Malley L (2005). Scoping studies: towards a methodological framework. Int J Soc Res Methodol.

[28] Covidence.

[29] Page MJ, McKenzie JE, Bossuyt PM (2021). The PRISMA 2020 statement: an updated guideline for reporting systematic reviews. BMJ.

[30] Birnbaum ML, Ernala SK, Rizvi AF (2019). Detecting relapse in youth with psychotic disorders utilizing patient-generated and patient-contributed digital data from Facebook. NPJ Schizophr.

[31] Nguyen VC, Lu N, Kane JM, Birnbaum ML, De Choudhury M (2022). Cross-platform detection of psychiatric hospitalization via social media data: comparison study. JMIR Ment Health.

[32] Birnbaum ML, Kulkarni PP, Van Meter A (2020). Utilizing machine learning on internet search activity to support the diagnostic process and relapse detection in young individuals with early psychosis: feasibility study. JMIR Ment Health.

[33] Zlatintsi A, Filntisis PP, Garoufis C (2022). E-prevention: advanced support system for monitoring and relapse prevention in patients with psychotic disorders analyzing long-term multimodal data from wearables and video captures. Sensors (Basel).

[34] Yan AY, Speed TJ, Taylor CO (2025). Relapse prediction using wearable data through convolutional autoencoders and clustering for patients with psychotic disorders. Sci Rep.

[35] Tsakmaki PV, Tasoulis S, Georgakopoulos SV, Plagianakos VP (2025). Reducing artifact preprocessing in heart rate variability–based personalized psychosis prediction using adaptive long short-term memory models. Int J Neural Syst.

[36] Adler DA, Ben-Zeev D, Tseng VWS (2020). Predicting early warning signs of psychotic relapse from passive sensing data: an approach using encoder-decoder neural networks. JMIR mHealth uHealth.

[37] Lamichhane B, Zhou J, Sano A (2023). Psychotic relapse prediction in schizophrenia patients using a personalized mobile sensing–based supervised deep learning model. IEEE J Biomed Health Inform.

[38] Zhou J, Lamichhane B, Ben-Zeev D, Campbell A, Sano A (2022). Predicting psychotic relapse in schizophrenia with mobile sensor data: routine cluster analysis. JMIR mHealth uHealth.

[39] Buck B, Scherer E, Brian R (2019). Relationships between smartphone social behavior and relapse in schizophrenia: a preliminary report. Schizophr Res.

[40] Mehrotra KG, Mohan CK, Huang H (2017). Anomaly Detection Principles and Algorithms.

[41] Pang G, Shen C, Cao L, Hengel AVD (2022). Deep learning for anomaly detection. ACM Comput Surv.

[42] Barnett I, Torous J, Staples P, Sandoval L, Keshavan M, Onnela JP (2018). Relapse prediction in schizophrenia through digital phenotyping: a pilot study. Neuropsychopharmacology.

[43] Henson P, D’Mello R, Vaidyam A, Keshavan M, Torous J (2021). Anomaly detection to predict relapse risk in schizophrenia. Transl Psychiatry.

[44] Cohen A, Naslund JA, Chang S (2023). Relapse prediction in schizophrenia with smartphone digital phenotyping during COVID-19: a prospective, three-site, two-country, longitudinal study. Schizophrenia (Heidelb).

[45] Meyer N, Joyce DW, Karr C (2022). The temporal dynamics of sleep disturbance and psychopathology in psychosis: a digital sampling study. Psychol Med.

[46] (2016). Smartphone ownership and internet usage continues to climb in emerging economies. https://www.pewresearch.org/global/2016/02/22/smartphone-ownership-and-internet-usage-continues-to-climb-in-emerging-economies/.

[47] Busk J, Faurholt-Jepsen M, Frost M, Bardram JE, Vedel Kessing L, Winther O (2020). Forecasting mood in bipolar disorder from smartphone self-assessments: hierarchical Bayesian approach. JMIR mHealth uHealth.

[48] Götzl C, Hiller S, Rauschenberg C (2022). Artificial intelligence-informed mobile mental health apps for young people: a mixed-methods approach on users’ and stakeholders’ perspectives. Child Adolesc Psychiatry Ment Health.

[49] Eisner E, Ball H, Ainsworth J (2025). Using passive sensing to predict psychosis relapse: an in-depth qualitative study exploring perspectives of people with psychosis. Schizophr Bull.

[50] Ribeiro MT, Singh S, Guestrin C "Why should I trust you?”: Explaining the predictions of any classifier.

[51] Lundberg SM, Lee SI A unified approach to interpreting model predictions. https://proceedings.neurips.cc/paper/2017/hash/8a20a8621978632d76c43dfd28b67767-Abstract.html.

[52] Gilpin LH, Bau D, Yuan BZ, Bajwa A, Specter M, Kagal L Explaining explanations: an overview of interpretability of machine learning.

[53] van Rossum MC, Vlaskamp LB, Posthuma LM (2022). Adaptive threshold–based alarm strategies for continuous vital signs monitoring. J Clin Monit Comput.

[54] Cvach M (2012). Monitor alarm fatigue: an integrative review. Biomed Instrum Technol.

[55] El Abdellati K, De Picker L, Morrens M (2020). Antipsychotic treatment failure: a systematic review on risk factors and interventions for treatment adherence in psychosis. Front Neurosci.

[56] Wang R, Wang W, Obuchi M On predicting relapse in schizophrenia using mobile sensing in a randomized control trial.

[57] Bergmeir C, Benítez JM (2012). On the use of cross-validation for time series predictor evaluation. Inf Sci.

[58] Roy Y, Banville H, Albuquerque I, Gramfort A, Falk TH, Faubert J (2019). Deep learning–based electroencephalography analysis: a systematic review. J Neural Eng.

[59] Gargari OK, Fatehi F, Mohammadi I, Firouzabadi SR, Shafiee A, Habibi G (2024). Diagnostic accuracy of large language models in psychiatry. Asian J Psychiatr.

[60] Liu J, Gu J, Tong M (2025). Evaluating the agreement between ChatGPT-4 and validated questionnaires in screening for anxiety and depression in college students: a cross-sectional study. BMC Psychiatry.

[61] Gutiérrez E, Quesada C, DeFraites E, Harper DJ, Mandavia AD (2025). Interpretable LLM-based detection of loose associations using synthetic speech data in early psychosis. Schizophr Bull.

[62] Xu W, Pakhomov S, Heagerty P (2025). Perplexity and proximity: large language model perplexity complements semantic distance metrics for the detection of incoherent speech. J Biomed Inform.

[63] European Health Data Space (EHDS) regulation. European Commission.

